# Bioactive Proteins and Peptides from *Pleurotus* spp. for Food and Human Health Applications—A Review

**DOI:** 10.3390/foods15193503

**Published:** 2026-09-30

**Authors:** Ehira Romero-Castelán, Juana Fernández-López, Mauricio Opazo-Navarrete, Jorge Lopez-Tellez, Jose A. Rodriguez, Irais Sánchez-Ortega, Paulo Cezar Bastianello Campagnol, Eva M. Santos

**Affiliations:** 1 Área Académica de Química, Universidad Autónoma del Estado de Hidalgo, Carretera Pachuca-Tulancingo km 4.5 s/n, Colonia Carboneras, Mineral de la Reforma, Hidalgo 42183, Mexico; ehira_romero@uaeh.edu.mx (E.R.-C.); lo319035@uaeh.edu.mx (J.L.-T.); josear@uaeh.edu.mx (J.A.R.); irais_sanchez5498@uaeh.edu.mx (I.S.-O.); 2 IPOA Research Group, Institute for Agri-Food and Agro-Environmental Research and Innovation, Miguel Hernández University (CIAGRO-UMH), Orihuela, 03312 Alicante, Spain; 3 Agriaquaculture Nutritional Genomic Center (CGNA), Temuco 4780000, Chile; mauricio.opazo@cgna.cl; 4 Núcleo de Investigación en Producción Alimentaria, Facultad de Recursos Naturales, Universidad Católica de Temuco, Rudecindo Ortega, Temuco 02950, Chile; 5 FoodTech SpA, Temuco 4780000, Chile; 6 Department of Food Science and Technology, Universidade Federal de Santa Maria, Santa Maria, Rio Grande do Sul CEP 97105-900, Brazil; paulo.campagnol@ufsm.br

**Keywords:** *Pleurotus* spp., protein, protein hydrolysate, peptide, bioactive, meat fortifiers, meat analog

## Abstract

Faced with the environmental and human health concerns associated with the production and consumption of meat and other traditional animal proteins, mushrooms have emerged as promising sources of sustainable high-quality proteins. The genus *Pleurotus*, which includes more than 30 edible species consumed worldwide, is highly valued for its delicious flavor and nutritional composition, above all its protein and bioactive peptide content. This review summarizes 52 original research articles published over the last 10 years about *Pleurotus* spp. proteins, protein hydrolysates, and peptides. It discusses protein extraction techniques and peptide separation methods, which influence their physicochemical properties and biological activities. Furthermore, the antimicrobial, antihypertensive, antioxidant, anti-cancer, hepatoprotective, immunomodulatory, and anti-inflammatory effects of proteins, protein hydrolysates, and peptides derived from *Pleurotus* spp. are analyzed. Finally, their potential applications in the food industry, including their use as flavor enhancers (such as umami compounds), functional ingredients, meat fortifiers and replacers, are described. Therefore, *Pleurotus* spp. proteins, protein hydrolysates, and peptides represent promising compounds with potential in the food field and human-health-related applications.

## 1. Introduction

The continuous growth of the global population is expected to increase the demand for high-quality proteins in the coming years, which is projected to double by 2050 [1]. Proteins are fundamental nutrients in human health, as they provide energy and support physiological functions including tissue growth, metabolic regulation, and immune responses [2]. Over human history, meat has been the principal source of protein. However, environmental and human health issues associated with the production and consumption of traditional animal proteins have driven research into sustainable and alternative protein sources such as plants, algae, and mushrooms [3].

Mushroom consumption has increased significantly in recent years. According to a Grand View Research report, the global mushroom market reached USD 71. 9 billion in 2025 and is projected to grow to USD 156.3 billion by 2033 [4,5]. Among the most relevant cultivated mushrooms, the genus *Pleurotus* is recognized as the second most widely cultivated mushroom group worldwide, after *Agaricus bisporus*. It comprises more than 30 species of global importance, including *Pleurotus ostreatus, Pleurotus eryngii, Pleurotus ferulae lanzi,* and *Pleurotus djamor*, among others [3,6]. Most *Pleurotus* species are edible basidiomycetes and are notable for their ability to grow on a wide variety of lignocellulosic agricultural and agro-industrial residues (such as bagasse, wood sawdust, straw, etc.) and to tolerate a broad growth temperature range, between 20 and 30 °C, making them suitable for cultivation under diverse environmental conditions [7,8,9]. In addition, species of this genus are characterized by their umami and delicate, unique taste, which contributes to their culinary value and increasing demand [3,6]. *Pleurotus* species are best known for their rich composition of nutrients, dietary fiber, minerals, and vitamins such as thiamine, riboflavin, nicotinic acid, biotin, ascorbic acid, and carotenoids. Moreover, these mushrooms produce a variety of bioactive proteins containing all essential amino acids [1,2,10].

Some authors have pointed out that *Pleurotus* fungi contain relatively high protein levels in their fruiting bodies, mycelia, and stipes, with 60–70% digestibility and exceptional quality [11]. These proteins include hydrophobins, glycoproteins, lectins, immunomodulatory proteins, proteins with ribosome-inactivating activity, among others [1,10,12,13]. These proteins exhibit functional properties such as emulsifying capacity, water-holding ability, and thermal stability, which enhance their potential applications in food systems, including meat products, meat analogs, bakery products, and protein-enriched foods [14,15]. However, their potential toxicological relevance and safety in edible *Pleurotus* spp. require further investigation [16].

Additionally, enzymatic hydrolysis of *Pleurotus* fungi proteins can release bioactive peptides with beneficial physiological activities such as antihypertensive, antioxidant, anti-inflammatory, anti-cancer, antimicrobial, and immunomodulatory effects [2,17,18,19]. Consequently, mushroom-derived proteins, protein hydrolysates, and peptides are increasingly being explored as promising ingredients for food and nutraceutical formulations that could enhance human health. This review aimed to analyze worldwide advances in the proteins and peptides from *Pleurotus* spp. field, their extraction and purification methods, and their applications in the food industry and human health over the last 10 years.

## 2. Literature Search and Bibliometric Analysis of Protein Hydrolysates and Peptides from *Pleurotus* spp.

Diverse species of *Pleurotus* mushrooms have been studied in the last 10 years to obtain bioactive proteins and/or peptides. This review integrates a classical bibliometric analysis. Data were searched on the ScienceDirect, Springer, and MDPI platforms, and the PubMed database, in January 2026. For this review, the following search strings were employed: 1: “*Pleurotus”* AND “protein” AND “bioactive”; 2: “*Pleurotus”* AND “peptide” AND “bioactive” in the title, abstract, or keywords to find papers published in the last 10 years. More than 2000 documents were initially recovered. The inclusion criteria included research published from 2017 to 2026, written in English, and reporting on proteins and/or peptides from *Pleurotus* spp. Repeated studies, as well as those that did not include *Pleurotus* spp. or works not providing information about proteins, protein hydrolysate, and peptides, were removed. Finally, 52 articles met the listed criteria and were used to construct Table 1.

**Figure 1:**
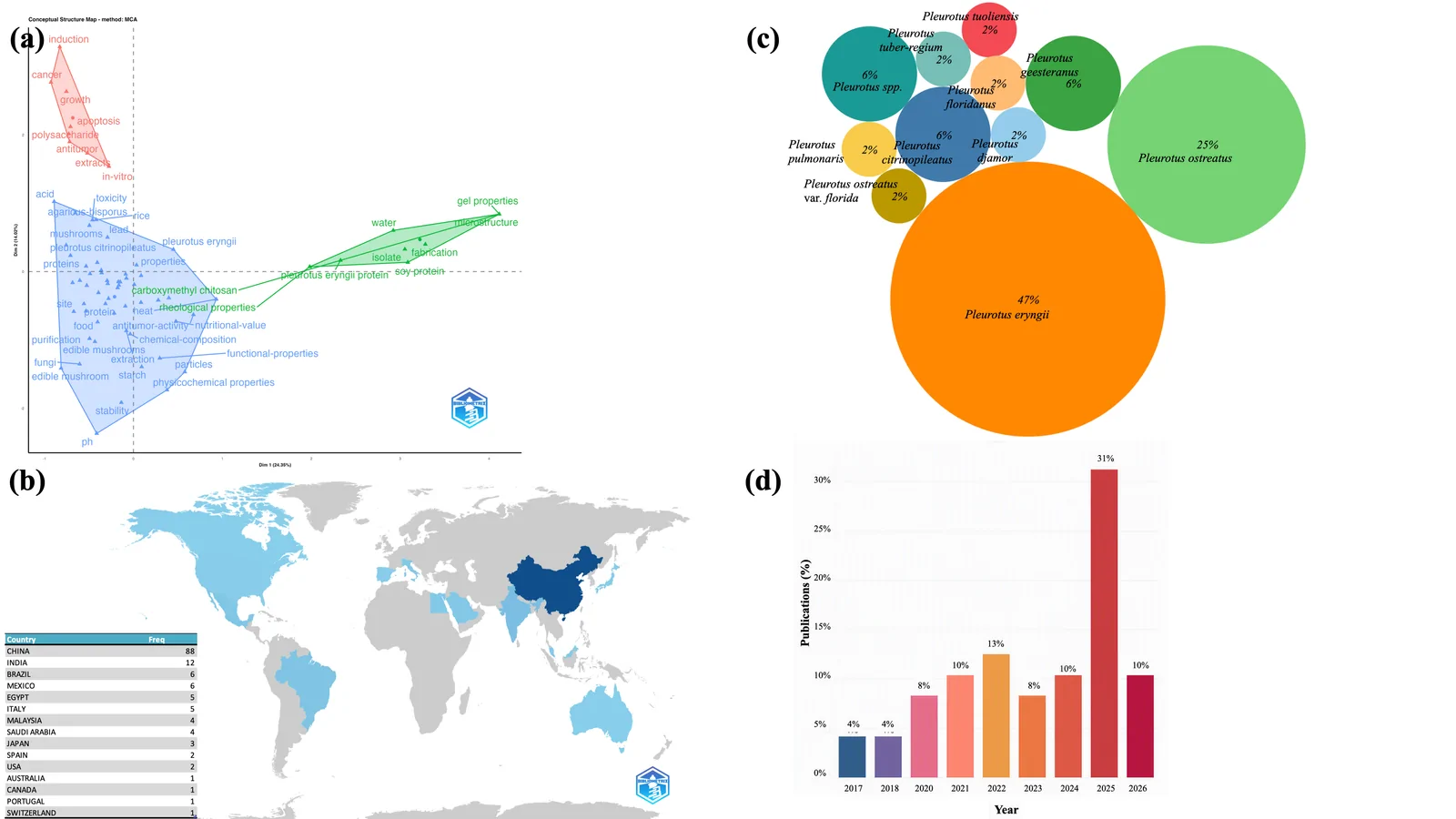
Bibliometric analysis of bioactive proteins and peptides from *Pleurotus* spp. (**a**) Conceptual structure of keywords of analyzed papers concerning bioactive proteins and peptides from *Pleurotus* spp. (**b**) Distribution by country of publication of papers on bioactive proteins and peptides from *Pleurotus* spp. (**c**) Distribution by *Pleurotus* species of publications on bioactive proteins, protein hydrolysates and peptides from *Pleurotus* spp. (**d**) Distribution by years of publications about bioactive proteins and peptides from *Pleurotus* spp.

A database with the 52 publications was also constructed using the Web of Science platform, and the bibliometric analysis was conducted in Biblioshiny from Bibliometrix (Figure 1) [20]. Figure 1a reflects the factorial analysis of keywords presented in the publications, divided into three clusters. The red cluster includes keywords related to the bioactivities of proteins and peptides from *Pleurotus* spp., such as antioxidant and antitumor activities. The green cluster encompasses keywords about technical properties, such as rheological properties. The blue cluster includes edible mushrooms and foods. The countries where more studies were conducted were China, India, Brazil, and México (Figure 1b). Figure 1c shows the distribution of the *Pleurotus* species used in the different publications, with *P. eryngii* being the most common, followed by *P. ostreatus* as a source for extracting bioactive proteins and peptides. Figure 1d presents the distribution of the analyzed publications by year, showing that 2025 was the year with the most published papers. According to the bibliometric analysis, the topic has not yet reached the maturity stage and is in the rapid growth phase, highlighting the interest in biological activities and food applications. In this work, some relevant references published before 2017 were used to provide background on the fundamental concepts of the study and to understand methodologies and/or mechanisms of action of *Pleurotus* spp. proteins, protein hydrolysates, and peptides. However, these references were not included in the bibliometric analysis. In addition, some relevant primary studies published within the analyzed period were identified outside the search strings and therefore were only used and cited for discussion purposes, without being included in the bibliometric dataset.

**Table 1:** Bioactivities and characteristics of proteins, proteins hydrolysates and peptides from *Pleurotus* spp. (2017–2026).

Pleurotus Species	Part of the Fungi	Product Type	Extraction Method	Purification Method	Molecular Weight (kDa)	Application	Results	Concept and Units of the Results	Ref.
*P. ostreatus*	Fruiting body	P	AlEx, EH, US-AEx and IP		NR	Food techno-functional properties	78.60–90.40 5.20–6.50 6.90–8.60 48.60–52.20 50.20–60.00 1.54–2.10 38.30–45.10	Protein solubility (%) Water holding capacity (g H_2_O/g) Oil holding capacity (g oil/g) Foaming capacity (%) Foaming stability (%) Emulsifying activity (m^2^/g) Emulsifying stability (min)	[1]
*P. eryngii*	Fruiting body	P	WEx, SO, OSP, AlEx	-	NR	Food techno-functional properties	807.90 1185.50 82.60 64.40 89.70 54.10 At 100 min digestion time: 85.00 At 100 min digestion time: 86.50	Water holding capacity (%) Oil holding capacity (%) Foaming capacity until (%) Foaming stability until (%) Emulsion activity index (%) Emulsion stability index (%) DPPH radical scavenging activity (%) ABTS radical scavenging activity (%)	[21]
*P. eryngii*	Fruiting body	P	AlEx and IP	Ultrafiltration	NR	Food techno-functional properties	8.00–18.00 13.00–25.00	Emulsifying activity index (m^2^/g protein) Emulsifying stability (%)	[22]
*P. eryngii*	Fruiting body	PS-P	AlEx	-	NR	Food techno-functional properties	At 1 Hz frequency: 60–180 At 1 Hz frequency: 7–20 At a shear rate of 0.1 s^−1^: 20–75	Elastic modulus (Pa) Viscous modulus (Pa) Apparent viscosity (Pa·s)	[23]
*P. eryngii*	Fruiting body	P	AlEx	Dialysis and CGP	39.63, 32.45 and 38.34	Food techno-functional properties	85–89	Water holding capacity (%)	[24]
*P. eryngii*	Fruiting body	P	AlEx	-	<1.00	Attenuate colitis in an in vivo mouse model	1.00–1.50 0.70–1.20 >50 >60 100–1300 0.5–6.5 125–1300	Colonic damage score reduction Colon length increase (cm) AST decrease (U/mg protein) ALT decrease (U/mg protein) Microbiota observed OTUs increase Microbiota Shannon diversity index increases Microbiota Chao diversity index increases	[25]
*P. eryngii*	Fruiting body	P	AlEx	-	NR	Food techno-functional properties	Gelation of myofibrillar protein		[26]
*P. eryngii*	Fruiting body	P	AlEx and IP	CGP	15.00, 35.00, 55.00 and 170.00	Lead detoxification activity in an in vivo lead-exposure mouse model	0.5	mg/kg Pb reduction in mouse brain-AE31	[27]
*Pleurotus geesteranus*	Fruiting body	P	AlEx	Dialysis	<45	Food techno-functional properties	At pH 6: 86.00 2.00 5.00 34.00 75.00 460.00 13	Protein solubility (%) Water-holding capacity (g/g) Oil-holding capacity (g/g) Foaming capacity (%) Foaming stability (%) Emulsifying activity (m^2^/g) Emulsifying stability (min)	[28]
*P. ostreatus*	Fruiting body	P	BEx and OSP		NR	Antimicrobial activity (in vitro)	Concentration 30 µg 20.50 19.00 27.00 19.53 18.60 21.76 19.83 17.66	Inhibition zone (mm) for: *Streptococcus mutans* *Streptococcus oralis* *Lactobacillus acidophilus* *Actinomyces viscosus* *Staphylococcus aureus* *Escherichia coli* *Enterococcus faecalis* *Candida albicans*	[18]
*P. eryngii*	Fruiting body	P	AlEx and IP	SDS-PAGE Electrophoresis	9.06, 1.69, 0.57 and 0.36	NR	NR	NR	[6]
*P. eryngii*	Fruiting body	P	AlEx and IP	-		Food techno-functional properties	0.90 60.00 72.85	Hardness (N) Water holding capacity (%) Elastic modulus (Pa)	[14]
*P. eryngii*	Fruiting body	P	AlEx and SO	CGP	14.68	Anti-cancer activity in an in vitro A549 cell model	Concentration of 200 µg/mL 24 h: 31.00 48 h: 64.00	A549 cell inhibition (%) A549 cell inhibition (%)	[29]
*P. eryngii*	Fruiting body	P	AlEx and IP	Dialysis and ultrafiltration	94.00, 52.00, 40.00, 37.00, 35.00, and 17.00	Food techno-functional and antioxidant activities	48.00–70.00 (pH 11) 75.00–92.00 (pH 10) 22.00–54.00 (pH 10)	Solubility (g/100 g) Emulsifying activity (g/mL) Emulsifying stability (min)	[30]
*P. eryngii*	Fruiting body	P	WEx and SO	SDS-PAGE Electrophoresis	17.5	Anti-cancer activity in an in vitro A549 cell model	Concentration of 200 μg/mL 25	Apoptosis in A549 lung cancer cells (%)	[17]
*P. ostreatus*	Fruiting body	P	Purchased	-		Antioxidant activity	85.79–88.39 69.20–96.48	DPPH scavenging activity (%) OH scavenging activity (%)	[12]
*P. geesteranus*	Fruiting body	P	AlEx and IP	Dialysis	<70.00	Food techno-functional properties	6.79 and 6.52 44.44 and 66.02	Oil holding capacity (g/g) Foaming capacity (%)	[31]
*Pleurotus tuber-regium*	Sclerotia	P	WEx and SO	Dialysis and CGP	84.20, 67.60 26.58 and 67.60	Anti-cancer activity in an in vitro MDA-MB-231 cell model	MDA-MB-231 cell IC_50_: 0.60–0.65	µg/mL	[32]
*Pleurotus tuoliensis*	Fruiting body	P	AlEx and IP	SDS-PAGE Electrophoresis	10.00–17.00, 26.00–34.00 and 43.00–55.00	Food techno-functional properties	52.30–285.89 88.82 109.35	Surface hydrophobicity Surface wettability (°) Oil-in-water contact angle (°)	[33]
*P. ostreatus* var. *florida*	Fruiting body	P	Foam extraction and OSP	CGP	PH: 13.53. PEP: 2.41, 2.39, 2.14, 1.57, 1.50 and 1.21	Hydrophobin	NR	NR	[34]
*P. ostreatus*	Fruiting body	P	WEx	EH	NR	Antioxidant, anti-cancer in an in vitro HeLa cell model and antihypertensive activities	Concentration of 10 mg/mL of protein with 65% hydrolysis degree: 84.00 88.50 90.75 84.70 19.19 2.31 67.00 87.00 12.73	DPPH radical scavenging activity (%) ABTS radical scavenging activity (%) Hydroxyl radical scavenging activity (%) Metal chelating activity (%) FRAP value (μM/mL) RPA (%) Linoleic acid oxidation inhibition day 3 (%) ACE inhibitory activity (%) HeLa cells antiproliferative activity (%)	[35]
*P. ostreatus*	Fruiting body	P	AlEx and IP	SDS-PAGE Electrophoresis	12.00, 13.00, 28.00, 32.00 and 35.00	Antioxidant activity	Total phenolic content: 24.20 DPPH IC_50_: 1.40 ABTS IC_50_: 15.00	mg GAE/g mg/mL mg/mL	[11]
*P. ostreatus*	Fruiting body	P	WEx and US-AEx and OSP		NR	Antioxidant activity	DPPH IC_50_: 39.46 ABTS IC_50_: 11.22 21.4 µg/mL	mg/mL mg/mL ORAC assay (μg/mL)	[36]
*P. ostreatus*	Mycelium	P	Foam extraction and OSP		10.00 and 17.00	Food techno-functional properties	80.90 57.90 43.50 19.04 14.40	Emulsification index in sunflower oil (%) Emulsification index in olive oil (%) Emulsification index in castor oil (%) Emulsification index in hexane (%) Decrease in contact angle of water (%)	[37]
*P. ostreatus*	Fruiting body	P	BEx and SO	CGP	14.26	Ribotoxin-like protein	NR	NR	[16]
*P. ostreatus*	Fruiting body	P	WEx and alkaline precipitation	EH and dialysis	3.00–5.00	Antioxidant and hepatoprotective activities (in vitro)	87.15 65.22 37.38 46.19	DPPH radical scavenging activity (%) H_2_O_2_ scavenging activity (%) LPO inhibition activity (%) Fe Ion chelating activity (%)	[38]
*P. eryngii*	Fruiting body	P	AlEx	EH	NR	Antioxidant activity	60 min: 41.00, 240 min: 70.00 60 min: 84.00, 240 min: 45.00 60 min: 39.00, 240 min: 84.00	Hydroxyl radical scavenging activity (%) DPPH radical scavenging activity (%) Superoxide anion radical scavenging activity (%)	[39]
*P. eryngii*	Fruiting body	P	AcREx and SO	CGP	21.90	Immunomodulatory activity in an in vitro macrophage model	Concentration of 200 μg/mL 90.00 60.00 15.00	IL-1β relative expression IL-6 relative expression TNF-α relative expression	[10]
*P. eryngii*	Fruiting body	P	AcEx and SO	CGP	40	Anti-inflammatory activity in an in vitro macrophage model	16.10–83.20 17.80–65.80 41.20–76.30 28.40–70.10 75.40 64.60	NO production levels decrease (%) IL-1β inhibition (%) IL-6 inhibition (%) Macrophage intracellular ROS decrease (%) p-IκB decrease (%) p-NF-κB p65 decrease (%)	[40]
*P. eryngii*	Fruiting body and mycelium	P	AlEx and IP	EH	0.71	NR	NR	NR	[41]
*Pleurotus* spp.	Fruiting body	PH	AlEx and IP	-	>30.00, 10.00–30.00, 5.00–10.00, 3.00–5.00 and <3.00	Anti-diabetic activity in an in vitro enzymatic model	31.65–72.48 2.99–62.12	α-amylase reduction activity (%) α-glucosidase reduction activity (%)	[42]
*P. eryngii* *P. ostreatus*	Fruiting body	PH	AlEx and IP	-	6.71 and 8.04	Food techno-functional properties	36.10 and 66.04 140.00 and 100.10 179.20 and 144.90 54.80 and 49.20 22.40 and 54.90 31.60 and 16.10	Emulsion stability index (min) Emulsion activity index (m^2^/g) Foaming capacity (%) Foam stability (%) Adsorbed protein o/w interface (%) Adsorbed protein air–water interface (%)	[43]
*Pleurotus* spp.	Fruiting body	PH	AlEx and IP	-	>30.00, 10.00–30.00, 5.00–10.00, 3.00–5.00 and <3.00	Antihypertensive activity in an in vitro ACE model	>30 kDa: 20.30 10–30 kDa: 20.60 5–10 kDa: 36.70 3–5 kDa: 48.50 <3 kDa: 38.50	% ACE inhibitory activity	[44]
*Pleurotus* spp.	Fruiting body	PH	AlEx and IP	SDS-PAGE Electrophoresis	>30.00, 10.00–30.00, 5.00–10.00, 3.00–5.00 and <3.00 kDa	Antioxidant and antihypertensive activities	“30 kDa”: 4.30, “10–30 kDa”: 15.40, “5–10 kDa”: 7.00, “3–5 kDa”: 38.80, “<3 kDa”: 4.90 “30 kDa”: 15.40, “10–30 kDa”: 5.60, “5–10 kDa”: 22.30, “3–5 kDa:” 100.00, “<3 kDa”: 39.60 “30 kDa”: 100, “10–30 kDa”: 88.00, “5–10 kDa”: 57.5, “3–5 kDa”: 60.00, “<3 kDa”: 50.00	ABTS scavenging activity (%) DPPH scavenging activity (%) FRAP (%)	[45]
*P. djamor* var. *salmoneostramineus*	Fruiting body	PH	AlEx and IP	EH	NR	In silico anti-angiogenic, anti-cancer, anti-inflammatory and anti-quorum sensing activities	Anti-angiogenic peptides: SQTTKESPSA and PQEDKSA Anti-cancer peptides: TSMQSSL, QEGQKL, SEDSGEA, and GRNSL. Anti-inflammatory peptides: TSMQSSL, TSQESY, QEGQKL Anti-quorum sensing peptides: TSMQSSL, PGSHPV, and GRNSL		[46]
*P. ostreatus* var. *florida*	Fruiting body	PH	OSP	Fermentation	<27.00	Anti-diabetic and antihypertensive activities in in vitro enzymatic models, and food technofunctional properties	α–amylase inhibitory activity IC_50_: 3.40–12.10 ACE-I inhibitory activity IC_50_: 0.01–5.70 8.53–19.50 15.03–23.30 127.21–145.16 145.66–157.23 292.28–340.13 126.78–144.9	mg/mL mg/mL Foaming capacity (%) Foaming stability (%) Emulsifying activity (%) Emulsifying stability (%) Water absorption capacity (%) Oil absorption capacity (%)	[47]
*Pleurotus citrinopileatus*	Fruiting body	PEP	WEx and US-AEx	CGP and SI	<3.00 and 3.00–10.00	Flavoring (in silico and human panel)	Umami peptides: LLVTVEDELRV, LDDWR, ENLLEK, and ELFEPT		[48]
*P. ostreatus*	Fruiting body	PEP	WEx and PEF-AEx	CGP and SI	NR	Anti-inflammatory activity (in silico)	Anti-inflammatory peptides:	WGATFGPY, GIETAGGVM, LPYDEFSAH, PIRWTPGSYP, DKEGIPPDQQRL and VPPDEIIKLE	[49]
*P. eryngii*	Fruiting body	PEP		EH, membrane filtration, CGP and SI	<3	Flavoring (in silico and human panel)	At a concentration of 1 mg/mL	AGHDDFP, GYDTF, and NGYDMR peptides intensified the perception of saltiness in a 0.3% NaCl solution	[50]
*P. eryngii*	Fruiting body	PEP	AlEx and IP	EH and CGP	0.90–2.00	Lead detoxification activity in an in vitro NCTC1469 cell model	Concentration of 320 µg/mL: 20.00–30.00	Pb treated NCTC1469 cells increase (%)	[51]
*P. ostreatus*	Fruiting body	PEP	WEx	CGP	NR	Flavoring (in silico and human panel)	LDFR peptide: umami, sourness, slight bitterness DRAFR peptide: umami, sourness AFDDALLK peptide: umami, sourness, sweetness VPLDDFR peptide: umami, sourness, astringency NGYDMR peptide: umami, sweetness VLRDF peptide: umami, sourness, slight bitterness		[52]
*P. eryngii*	Stipe	PEP	WEx and US-AEx	CGP and SI	NR	Anti-senescence activity (in vitro)	Concentration 1.0 mg/mL 51.53 39.86 Extensive alterations in differentially expressed genes between Model and Control, therefore it was established a senescence model	ROS generation reduction (%) SA-β-gal-positive cells reduction (%)	[53]
*P. eryngii*	Fruiting body	PEP		CGP and SI	<3.50 and 3.50–8.00	Antioxidant activity	Concentration of 50 mg/mL “0–3.50 kDa”: 37.40, “3.50–8.00 kDa”: 34.00 “0–3.50 kDa”: 0.50, “3.50–8.00 kDa”: 0.35 Concentration of 10 mg/mL “0–3.50 kDa”: 75.70, “3.50–8.00 kDa”: 46.60	DPPH radical scavenging activity (%) FRAP (μmol/mL) ABTS radical scavenging activity (%)	[54]
*P. eryngii*	Stipe	PEP	AlEx	EH and dialysis	<1	Anti-senescence activity (in vitro)	“Delayed D-galactose-induced senescence in PC12 cells by modulating the TLR4/NF-κB/MAPK signaling pathway”		[55]
*P. eryngii*	Fruiting body	PEP		EH and CGP	<5.00, 5.00–10.00, >10.0	Lead detoxification activity in an in vitro NCTC1469 cell model	Concentration 0.6 mg/mL: “<5 kDa”: 26.00, “5–10 kDa”: 2.00, “>10 kDa”: 3.00	Injured NCTC1469 viability increase (%)	[56]
*P. eryngii*	Fruiting body	PEP	OSP	Ultrafiltration and CGP	NR	Ribosomal peptides	NR	NR	[57]
*P. geesteranus*	Fruiting body	PEP	WEx	EH and dialysis	<10.00	Flavoring	“Enhance taste”		[58]
*P. ostreatus*	Fruiting body	PEP and AA	WEx and US-AEx and OSP	CGP	NR	NR	NR	NR	[59]
*P.citrinopileatus*	Fruiting body	PS-PEP	WEx and OSP	CGP	16.08–1216.00	Hepatoprotective activity (in vitro)	Concentration 40 µg/mL: 130.00–303.00 110.00–220.00 3046.00–5785.00 2218.00–4927.00 3.29–4.89	HepG2 injured cell viability (%) Intracellular TG reduction (µg/mL) ALT reduction (U/mg) AST reduction (U/mg) T-SOD increase (U/mg)	[60]
*P. ostreatus*	Mycelium	PS-PEP	BEx and OSP	CGP	NR	Hepatoprotective activity in rats treated with DEN/CCL_4_	Concentration of 1 μg/mL 208.30 199.67 70.83 69.54	ALT reduction (U/L) AST reduction (U/L) ALP reduction (U/L) LPO reduction (U/L)	[61]
*P. eryngii*	Mycelium	PEP	BEx	CGP	NR	Antioxidant, anti-cancer in cell models and immunostimulatory activities in in vitro models	Concentration of 1 mg/mL F1: 45.0, F2: 60.1 F1: 38.0, F2: 49.0 F1: 49.0, F2: 83.0 F1: 43.0, F2: 52.0 F1: 69.0, F2: 87.0 F1: 225.0, F2: 125.0 F1: 8.0, F2: 2.0 F1: 4.0, F2: 2.5	Hydroxyl radical scavenging activity (%) DPPH radical scavenging activity (%) BT-549 cell viability (%) HeLa-229 cell viability (%) HGC-27 cell viability (%) TNF-α increase (ng/mL) TLR2 increase (ng/mL) TLR4 increase (ng/mL)	[62]
*Pleurotus pulmonarius*	Mycelium	PEP	WEx and SO	CGP	PH: 63.00, 55.00 and 11.00. PEP: 0.33, 0.37, 0.39 and 0.33	Antihypertensive activity in an in vitro ACE model	ACE IC_50_: 55–110	μg/mL	[63]

PEP: peptide, AA: amino acids, PS-PEP: polysaccharide–peptide, PS-P: polysaccharide–protein, P: protein, PH: protein hydrolysates, AcEx: acid extraction, SO: salting out, AcREx: acid reductive extraction, AlEx: alkaline extraction, IP: isoelectric precipitation, US-AEx: ultrasound-assisted extraction, BEx: buffer extraction, OSP: organic solvent precipitation, EH: enzymatic hydrolysis, WEx: water extraction, PEF-AEx: pulsed electric field assisted extraction, CGP: chromatographic purification, SI: spectrometric identification, and NR: not reported.

## 3. Protein Extraction Methods from *Pleurotus* spp.

The extraction of proteins from species of the genus *Pleurotus* has gained increasing attention. However, extraction methods strongly influence protein efficiency and quality by determining protein yield, structure, and functional properties [53,64].

**Figure 2:**
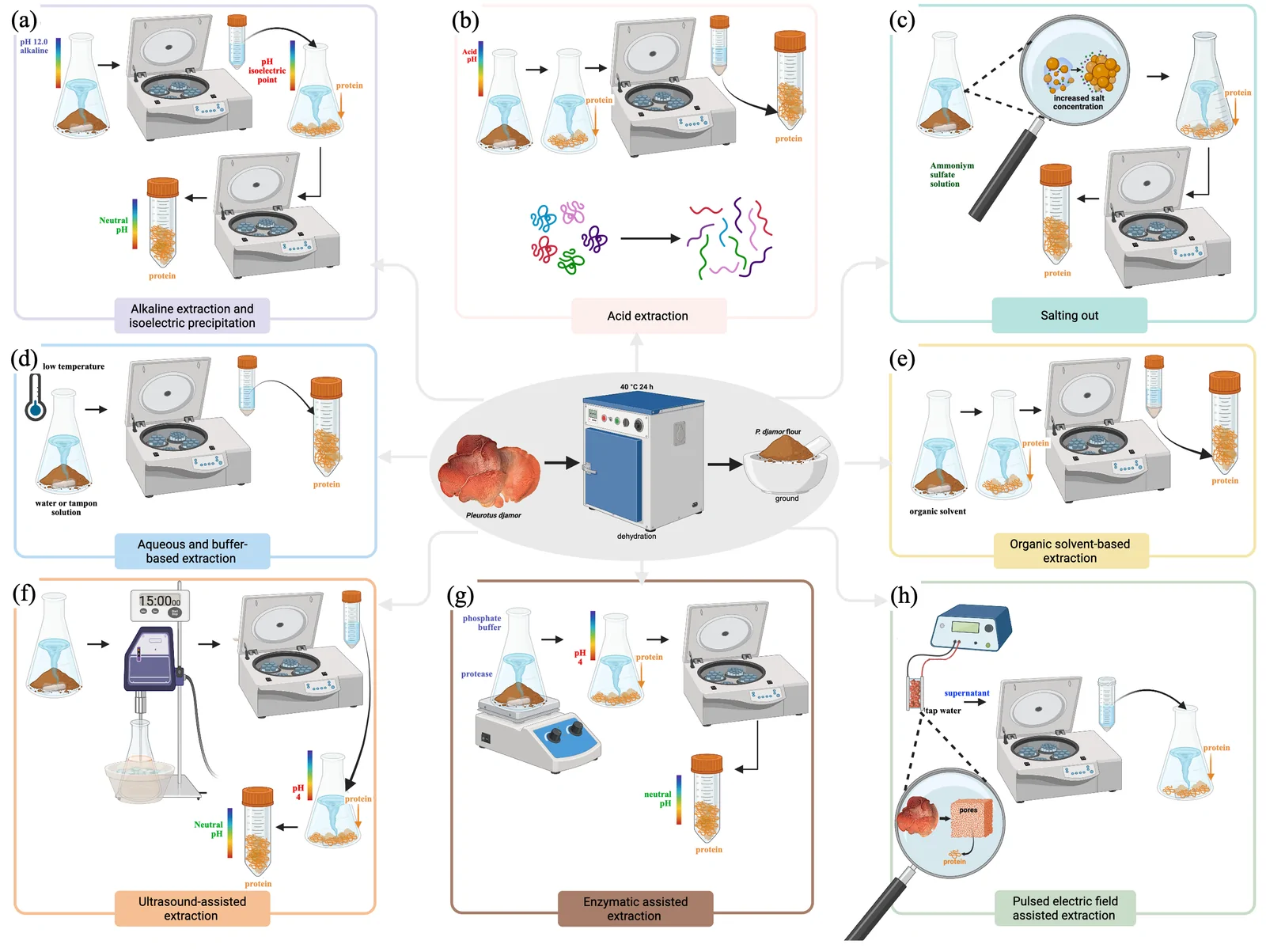
Extraction methods to obtain proteins from *Pleurotus* spp.: (**a**) alkaline extraction and isoelectric precipitation extraction method; (**b**) acid extraction method; (**c**) salting out; (**d**) aqueous and buffer-based extraction method; (**e**) organic solvent-based extraction method; (**f**) ultrasound-assisted extraction method; (**g**) enzymatic-assisted extraction method; (**h**) pulsed electric field-assisted extraction method [1,42,49,65,68]. Created in BioRender. Romero-Castelán, E. (2026) https://BioRender.com/hj3d24j.

Prior to protein extraction techniques, dehydration, grinding, and defatting steps are essential to reduce the particle size and increase the surface area to yield better extraction efficiency. Alkaline protein solubilization at basic pH (around 12), followed by isoelectric precipitation (AlEx and IP), is the commonly used technique applied for protein extraction in *Pleurotus* spp. (Figure 2a) due to its simplicity and high efficiency [33,38,65]. In contrast, acid extraction is less commonly used and employs an acidic solution to solubilize proteins or to partially or completely hydrolyze amino acids before subsequent purification steps (Figure 2b) [40,66]. Nevertheless, these techniques can induce protein denaturation and irreversible structural changes under extreme pH conditions, reducing protein solubility and functionality [31,53].

Salting out (SO) (Figure 2c) is an alternative method where a high salt concentration is added to the protein suspension (typically ammonium sulfate). This increases the interactions between water and salt ions; as a result of the reduced availability of water, protein–protein interactions occur, then proteins aggregate and precipitate, better preserving their native conformation without large unfolding [31,62,63]. In addition to conventional techniques, aqueous and buffer-based extraction methods (WEx and BEx) consist of homogenization by agitation of mushroom powders in distilled water or buffers (such as phosphate or Tris–HCl) under cold conditions, followed by centrifugation, where the supernatant containing soluble proteins is recovered. These techniques preserve protein structure and functionality, but lower extraction yields are obtained compared to alkaline methods (Figure 2d) [61,62,63,67]. Precipitation with organic solvents (OSP) involves adding ethanol, acetone, or trichloroacetic acid (TCA) to reduce protein solubility, leading to protein aggregation and precipitation to recover total proteins, or after enzymatic hydrolysis to fractionate peptides (Figure 2e) [34,37].

In recent years, emerging technologies have been explored to avoid the limitations of conventional methods. For instance, the ultrasound-assisted extraction method (US-AEx) (Figure 2f) consists of treating the defatted protein suspension with an ultrasonicator for at least 15 min in an ice bath to prevent protein heating. The supernatant is collected, and the pH is reduced to 4 in order to precipitate protein [1]. US-AEx has shown superior extraction efficiency, which could be attributed to acoustic cavitation phenomena that facilitate solvent penetration and protein release [53]. Furthermore, the enzyme-assisted extraction method (Figure 2g) consists of suspending mushroom flour in a phosphate buffer containing a protease enzyme. The enzymatic treatment promotes the degradation of cell wall components and associated molecular complexes, thereby enhancing protein availability. However, these techniques may present challenges related to process cost, scalability, and some alterations in protein structure [1,53]. Among novel methods, salt solubilization assisted by ultrasound has emerged as a promising strategy, implying a mild extraction that preserves protein structure and functionality. Pulsed electric field-assisted extraction (PEF-AEx) (Figure 2h) has been used on fresh crushed *Pleurotus* spp. at a conductivity between 600 and 800 μS/cm, applied energy per kg of sample of 50 kJ, electric field strength between 2.5 and 10 kV/cm, electric pulse duration of 100 ms and frequency of 2.00 Hz, with specific parameters reaching a temperature of 30 °C, to create pores in the cells and allow proteins to be released [49]. Calleja-Gómez et al. [49] showed that for *P. eryngii* protein extraction, PEF-AEx does not induce protein fragmentation or peptide chain cleavage, as evidenced by unchanged SDS-PAGE profiles compared to untreated samples. However, PEF-AEx may promote protein conformational changes, expose internal functional groups, and form higher-molecular-weight aggregates [49].

As shown in Table 2, the method selected for *Pleurotus* spp. protein extraction can influence protein structure and its extraction yield. Although alkaline extraction with isoelectric precipitation uses extreme pH conditions, FTIR analysis shows it preserves nearly all native α-helical secondary structure; however, isoelectric precipitation compromises protein solubility by exposing hydrophobic residues on the surface. In contrast, ultrasound-assisted extraction induces a dose-dependent loss of secondary structure, with the highest-yielding condition also producing the greatest structural disruption, including partial peptide-bond cleavage and increased protein unfolding [1,65,68]. Enzyme-assisted extraction can preserve native secondary structure through selective enzymatic cleavage at a lower yield [1,65,68]. Changes in protein conformation, solubility, and accessibility may alter enzyme–substrate interactions, thereby affecting the degree of hydrolysis and the molecular size, sequence, and functional properties of the resulting peptides. Therefore, extraction efficiency is not the principal criterion for method selection; the preservation of protein characteristics and the requirements of downstream hydrolysis and the intended food application must also be considered.

**Table 2:** Comparison between the *Pleurotus* spp. protein extraction methods [1,42,49,65,68].

Extraction Method	Advantage	Disadvantages
Alkaline extraction + isoelectric precipitation	Simple, efficient solubilization, low-cost and widely validated, minimal secondary-structure disruption	Irreversible protein unfolding and exposure of hydrophobic side chains, protein losses during isoelectric precipitation
Acid extraction	Low-cost, useful for partial hydrolysis, low molecular weight peptide formation, and simple	Mineral acids induce irreversible denaturation
Salting out	Preserves native conformation and original molecular weight, moderate cost	Requires salt removal and additional processing
Aqueous/buffer extraction	Mild conditions, relatively simple and may preserve functionality of proteins and peptides	Lower extraction yield reported in some studies
Organic-solvent precipitation	Useful for protein recovery/fractionation and high protein yield	Solvent removal required, can cause segregation and regulatory restrictions
Ultrasound-assisted extraction	Highest protein recovery and yield, enhances solvent penetration and protein release, and lowest carbohydrate co-extraction	High energy requirements, may cause dose-dependent structural disruption at high intensity or prolonged treatment duration, increased browning
Enzyme-assisted extraction	Enhances protein release through degradation of cell-wall components, higher selectivity, preserves native secondary structure via selective cleavage, low molecular weight peptide formation	Enzyme cost, low yield due to formation of soluble peptides that do not precipitate, and protein browning
Ultrasound-assisted salt solubilization	Mild conditions and high efficiency; may improve protein release while preserving functionality	Limited validation and may cause heating and dose-dependent structural disruption at high intensity or duration
Pulsed electric field	Non-thermal cell degradation, facilitates protein release, does not fragment proteins, nor cause chain breaks	Costly, partially unfolds proteins, poorly validated and requires complex optimization

## 4. Protein Hydrolysates and Peptides from *Pleurotus* spp. Proteins

Following extraction, ultrafiltration is used to separate protein fractions based on molecular weight cut-off, resulting in peptides and proteins of different size ranges. Dialysis, applied to remove salts and solvents, could also be employed to separate different molecular weight protein fractions [54,55,69]. On the other hand, ion-exchange chromatography, such as DEAE- or CM-Sephadex columns, separates proteins based on charge, while gel filtration chromatography (e.g., Sephadex G-75 or G-100) separates molecules according to size [10,70,71]. Additionally, reverse-phase high-performance liquid chromatography (RP-HPLC) can be used for further purification of specific protein or peptide fractions [51,55,63].

In addition, *Pleurotus* proteins could be hydrolyzed by microbial fermentation, mainly by lactic acid bacteria (LAB) at pH 6 and 37 °C; with proteolytic systems, LAB catalyze the protein bonds into peptides of lower molecular weight (MW) [19,47,72]. Zahoor et al. [47] employed *Lactobacillus acidophilus* and *Lactobacillus brevis* to hydrolyze *P. ostreatus* var. *florida* protein, obtaining peptides with molecular weights below 27 kDa and increasing the foaming capacity from 8.53% to 19.50% after fermentation. Furthermore, ACE-inhibitory activity was enhanced after fermentation, with the IC_50_ decreasing from 5.70 to 0.01 mg/mL.

Enzymatic hydrolysis plays a crucial role in the generation of bioactive protein hydrolysates and peptides. When this process simulates gastrointestinal digestion of *Pleurotus* proteins, the generated peptides are typically composed of 7–15 amino acids. Enzymatic hydrolysis is highly dependent on operational parameters such as protease type (alone or in combination), temperature, pH, and hydrolysis time, which influence the degree of hydrolysis and the resulting protein hydrolysate and/or peptide profile [53,64]. Enzymatic hydrolysis is considered a green and efficient approach, enabling controlled cleavage of peptide bonds and the production of peptides with specific molecular weights and biological activities, unlike microbial fermentation. Notably, low-molecular-weight peptides, particularly those enriched in hydrophobic and charged amino acids, have been associated with enhanced bioactivities, such as antioxidant and antihypertensive effects, compared to their parent proteins [46,64].

## 5. Bioactivities and Applications of Proteins, Protein Hydrolysates and Peptides of *Pleurotus* spp.

### 5.1. Antioxidant Activity of Bioactive Protein Hydrolysates and Peptides from Pleurotus spp.

Protein hydrolysates and peptides derived from *Pleurotus spp*. have exhibited antioxidant activity, regulated by factors such as molecular weight, amino acid composition, and transformation of mushroom conditions [21,30,35,39,43,62,67]. Low MW fractions (<5 kDa) typically show higher antioxidant capacity [21,30,35,39,43,62,67]. These antioxidant mechanisms are mainly linked to the neutralization of reactive oxygen species (ROS), as evidenced by assays such as 2,2-diphenyl-1-picrylhydrazyl (DPPH•), 2,2-azino-bis-3-ethylbenzothiazoline-6-sulphonic acid (ABTS), superoxide (O_2_•^−^), and hydroxyl radicals (•OH), as well as metal chelation [43,54,67].

Hydrophobic amino acids such as Ala, Val, Leu, Ile, and Met enhance antioxidant activity since they possess numerous electrons that could be donated to free radicals, while aromatic and polar amino acids like Tyr, Trp, Phe, and His contribute to proton and electron donation, stabilizing free radicals [21,55]. Additionally, negatively charged amino acids such as Asp and Glu improve metal-chelating capacity by providing binding sites for metal ions like Fe^2+^, thus preventing oxidative reactions [30]. Furthermore, antioxidant capacity is regularly concentration-dependent [30]. Processing methods could modulate antioxidant activity since modifications in proteins and/or peptides could alter their structure or might lead to the formation of smaller peptides with different antioxidant mechanisms, maintaining or even enhancing the activity [21].

Wang et al. [21] obtained proteins from *P. eryngii* with high antioxidant activity according to DPPH• and ABTS activity. This activity increased when proteins were subjected to simulated gastric digestion, resulting in their fragmentation into hydrolysates, peptides, and free amino acids in a time-dependent manner, with a limit of 120 min, after which a decline was observed due to the degradation of antioxidant components by gastric enzymes. Hou et al. [43] obtained hydrolysates from *P. eryngii* and *P. ostreatus* protein of 6.7 and 8.04 kDa, respectively, which exhibited a Fe^2+^ chelating activity of more than 10%, scavenging activity against DPPH• of more than 45% and against hydroxyl radical of more than 30%, in a concentration-dependent manner. These authors found that when hydrolysates were subjected to enzymatic hydrolysis, the antioxidant activity increased significantly, which was attributed to the release and increased exposure of hydrophobic amino acid residues during enzymatic hydrolysis. Wu et al. [30] isolated proteins from *P. eryngii* of different molecular weights that showed an O_2_•^−^ scavenging capacity of 10–40%, a Fe^2+^ chelating force of 35–70%, a DPPH• scavenging activity of 10–20%, and an •OH scavenging capacity of 35–80%. They found that low-molecular-weight proteins showed higher activities in a concentration-dependent manner because they can donate amino groups more readily to stabilize free radicals. Goswami et al. [35] reported a similar behavior in protein hydrolysates derived from *P. ostreatus,* which exhibited superior metal chelating activity (84.70%), ferric reducing antioxidant power (FRAP) (19.19 μM/mL), DPPH• scavenging activity (84.00%), ABTS• radical scavenging activity (88.50%), and •OH scavenging activity (90.75%) when enzymatic hydrolysis reached 65% degree. Antioxidant activities reported in these studies were measured using chemical assays that quantify radical-scavenging capacity under artificial, non-physiological redox conditions and do not necessarily reflect real protection against oxidative stress in living cells or tissues.

### 5.2. Immunomodulatory and Anti-Inflammatory Effects of Bioactive Protein Hydrolysates and Peptides from Pleurotus spp.

*Pleurotus* spp. derived proteins and peptides have demonstrated immunomodulatory activity by stimulating defense cells such as macrophages and dendritic cells, which could mitigate systemic chronic inflammation [10,40,62,67,73]. In order to maintain homeostasis, macrophages can polarize into two distinct functional phenotypes, depending on the activating stimuli: M1 and M2 [10,74]. The M1 phenotype represents the pro-inflammatory or activated state, initiated by interferon-γ (IFN-γ) or lipopolysaccharide (LPS); this activation is beneficial for the enhancement of innate defense mechanisms. The cytokines produced during M1 polarization, including TNF-α, interleukins IL-1β, IL-6, IL-12 and chemokines CCL2, CXCL1, and CXCL10, are regulated by the nuclear factor kappa-light-chain-enhancer of activated B cells complex (NF-κB) and mitogen-activated protein kinase pathways (MAPK), two intracellular signaling pathways that regulate the inflammatory response [10,40,74]. M2 phenotype, induced by cytokines IL-4 or IL-13, is associated with anti-inflammatory responses and tissue repair, producing IL-8 and IL-10 cytokines [10,74]. For example, Hu et al. [10] found that a protein from *P. eryngii* of 21.9 kDa induced M1 polarization in RAW 264.7 macrophage cells, stimulating the production of IL-1β, IL-6, TNF-α, and nitric oxide (NO), an agent that can destroy pathogens and tumor cells, through iNOS expression enhancement in a dose-dependent manner. In another work, Sun et al. [62] also found an increase in NO activity and TNF-α and IL-6 secretions in macrophage Ana-1 cells when treated with a polypeptide from *P. eryngii*. In addition, this polypeptide induced a dose-related increase in transmembrane Toll-like proteins (TLR 2 and TLR 4), which are important in pathogen recognition.

On the other hand, chronic inflammation is a systemic response linked to diseases such as cancer or diabetes, cardiovascular problems, among others [75,76]. In this case, modulation of the M2 phenotype is required to reduce systemic chronic inflammation and suppress inflammatory responses [40]. Yuan et al. [40] isolated a protein of 40 kDa that produced a reduction in the activation of NF-κB and MAPK signaling when 50 μg/mL, 100 μg/mL and 200 μg/mL solutions of the protein were used, showing concentration-dependent inhibition of IL-1β and IL-6 ranging from 17.80 to 65.80% and from 41.20 to 76.30%, respectively. Thus, the proteomic diversity of *Pleurotus* spp. allows it to function as a homeostatic agent, capable of either stimulating cellular immunity to establish an effective defense or suppressing excessive inflammatory responses to maintain physiological balance, depending on the specific bioactive protein involved and the host’s biological requirements. However, no study has been conducted in a clinical trials to confirm whether these effects persist after simulated gastrointestinal digestion. Therefore, further in vivo and trial studies are required.

### 5.3. Antihypertensive Activity of Bioactive Proteins, Hydrolysates and Peptides from Pleurotus spp.

According to Zhang et al. [76], the consequence of chronic inflammation, a dysregulated immune system, and oxidative stress could be hypertension, the precursor of most cardiovascular diseases. When immune cells, such as monocytes, neutrophils, dendritic cells, and M1 macrophages, promote the pro-inflammatory response, cytokines and chemokines generate vascular inflammation and endothelial dysfunction [76]. Cytokines and interleukins (such as IFN-γ and IL-17) contribute to oxidative stress and vascular damage, enhancing the generation of ROS, which act as mediators relating immune responses to hypertension [76,77]. Additionally, activation of the renin–angiotensin–aldosterone system occurs through the angiotensin-converting enzyme (ACE), which catalyzes the conversion of angiotensin I into angiotensin II, a potent vasoconstrictor. ACE increases oxidative stress by NADPH oxidases (NOX), intensifying inflammation, endothelial dysfunction, and vascular remodeling [76,78].

Bioactive protein hydrolysates and peptides derived from *Pleurotus* species present potential as natural antihypertensive agents since they have shown ACE inhibitory activity [44,63,67,79]. The ACE inhibitory activity of these protein hydrolysates and peptides is influenced by their structural characteristics, particularly molecular weight, amino acid sequence, and composition [44,67]. Low-molecular-weight protein hydrolysates (<10 kDa) and peptides (<3 kDa) exhibit higher inhibitory activity due to their accessibility and affinity for the active site of ACE, preventing the conversion of angiotensin I into angiotensin II [44,67]. Furthermore, the presence of hydrophobic amino acids (Pro, Val, Leu, Ile, Phe, Tyr, and Trp) and basic amino acids (Arg and Lys) in the C-terminal region, or/and aliphatic amino acids at the N-terminal (Val, Leu, and Ile), enhances the hydrophobic and electrostatic interactions between the hydrolysate proteins or peptides and the ACE active site [63,67].

In addition, ACE inhibition occurs through competitive binding to its active site, a zinc-dependent catalytic domain composed of the subsites S1, S2, and S1’ [44,80]. Bioactive protein hydrolysates and peptides from *Pleurotus* can interact with these regions by electrostatic forces, hydrogen bonds, and hydrophobic interactions, reducing ACE activity [44,80]. The sequence and positioning of amino acids in the last three residues of the C-terminal are determinants of inhibitory efficiency, since S1 and S1’ recognize hydrophobic and basic amino acids, while S2 contributes to the stability of the interaction when aliphatic amino acids are located near the N-terminal [44,80]. Chen et al. [67] developed a cold water extract from *P. citrinopileatus* and generated four fractions with a high protein content and molecular weights of less than 1 kDa, 1–3.5 kDa, 3.5–10 kDa and more than 10 kDa, respectively. These fractions showed ACE inhibition from 36.9% to 53.1%. They also found that the fraction with a molecular weight higher than 10 kDa showed an IC_50_ of 2.4 mg/mL. In another study, Lima de Carvalho Cavenaghi et al. [44] obtained isolated protein hydrolysates from *Pleurotus* spp. with molecular weights of 3 and 5 kDa that showed 48.50% ACE inhibition. The authors reported that LPILP, IPLLP, PLLPQ, and VIQYDPPQ peptides presented more ACE inhibition activity due to the presence of hydrophobic amino acids in the C-terminal portion and aliphatic amino acids in the N-terminal. Furthermore, Manoharan et al. [63] obtained peptides from *P. pulmonarius,* with the GVR peptide being the most anti-ACE active, with an IC_50_ of 55 μg/mL; likewise, they found that the binding site of ACE with GVR was at the center of the protein mediated by hydrogen bonds. Although *Pleurotus* protein hydrolysates and peptides exhibit ACE inhibitory activity, their effect remains considerably lower than the activity of the captopril drug, whose IC_50_ is in the nanomolar range, compared to the 55 μg/mL IC_50_ of the tripeptide derived from mycelium of *P. pulmonarius* reported by Manoharan et al. [63].

### 5.4. Anti-Cancer Effects of Bioactive Proteins, Hydrolysates and Peptides from Pleurotus spp.

As mentioned before, protein hydrolysates and bioactive peptides enhance antioxidant activity by neutralizing reactive oxygen species (ROS) and inhibiting their formation, thereby disrupting oxidative stress-mediated signaling pathways involved in tumor formation. In this context, mitochondrial regulation of redox balance plays a critical role, particularly through proteins such as uncoupling protein-2 (UCP2) [46]. This protein, located in the inner mitochondrial membrane, is an anion carrier that participates in proton transport via fatty acid cycling, contributing to the dissipation of the mitochondrial proton gradient [46]. Through these mechanisms, UCP2 modulates mitochondrial function, cellular metabolism, and ROS production. Some authors have reported that UCP2 values are elevated in several cancers such as pancreas, breast, leukemia, colon adenocarcinoma, etc. [46,81,82]. Consequently, targeting UCP2 in combination with conventional treatments could restore oxidative stress levels and might weaken cancer cells prior to treatment [46]. Ventura-García et al. [46] evaluated some *P. djamor* peptides in an in silico study using the PeptideRanker tool and selected eight peptides: SQTTKESPSA, PQEDKSA, TSMQSSL, TSQESY, QEGQKL, SEDSGEA, PGSHPV and GRNSL. The authors used PepSite2, MDockPeP, and HPEPDOCK 2.0 to measure the in silico UCP2 inhibitory activity of peptides by molecular docking, finding that all peptides could have feasible binding with UCP2 and suppress it. They also found that SEDSGEA and SQTTKESPSA peptides could have an effect against breast and prostate cancer cells, respectively, while PQEDKSA, TSMQSSL, PGSHPV, and GRNSL TSQESY peptides may have activity against colon cancer cells, and QEGQKL was related to activity against skin cancer cells. Moreover, the authors reported that *P. djamor* protein hydrolysate reduced human Hepatoma G2 (HepG2) cell viability in a dose- and time-dependent manner.

On the other hand, *Pleurotus* spp. peptides could have an effect on the stimulation of AMP-activated protein kinase (AMPK), which acts as an energy sensor and negatively regulates the mammalian target of rapamycin (mTOR), a central regulator of cell growth and metabolism [29,46]. Downregulation of mTOR promotes autophagy, a process that can lead to cancer cell death [29]. This is accompanied by increased expression of proteins that are also related to autophagy, such as LC3-II, Beclin-1, ATG7, and DRAM [29]. For instance, Liu et al. [17] isolated a protein from *P.eryngii* var. *ferulae* of 17.5 kDa with an in vitro anti-non-small cell lung cancer (NSCLC) A549 activity due to the mitochondrial apoptosis pathway, which is characterized by the upregulation of pro-apoptotic proteins such as BAX and BID, and the activation of the enzyme caspase-9 and PARP cleavage process. Likewise, Liu et al. [29] isolated another protein from the same mushroom of 14.68 kDa and 135 amino acid residues that showed upregulation of AMPK in NSCLC A549, the suppression of mTOR, and the upregulation of P62, LC3 II/I, P53, and P21 protein expression, inducing cell cycle arrest at the G1 phase. Furthermore, these authors observed a reduction in lung cancer tumor weight when mice were treated with this fungal peptide designated as PFAP, since the amino acid residues of these peptides could bind to receptors on cancer cells or tumor vasculature. As mentioned before, *Pleurotus* spp. derived proteins can modulate the immune response by activating dendritic cells through NF-κB and MAPK signaling pathways, enhancing antitumor protection [40,55].

### 5.5. Hepatoprotective Effects of Bioactive Proteins, Hydrolysates and Peptides from Pleurotus spp.

Inflammation, oxidative stress, and dysregulation of the renin–angiotensin system can activate NADPH oxidases and ROS, contributing to hepatic inflammation, fibrosis, and non-alcoholic fatty liver disease, which are generally diagnosed based on elevated serum levels of alanine aminotransferase (ALT) and aspartate aminotransferase (AST) [60,83]. In this context, bioactive peptides from species belonging to the genus *Pleurotus* could reverse elevated ALT/AST levels, offering a complementary strategy to drugs to manage metabolic liver disorders [60,61]. Abdel-Monem et al. [61] found an exopolysaccharide–peptide complex from *P. ostreatus* with protective effects against alterations induced by the hepatotoxic agents DEN/CCl_4_ in rats. When injured rat livers were treated with the complex, ALT and AST were restored almost to normal levels. Moreover, white blood cell and red blood cell counts of injured rats increased, as well as hemoglobin levels. Additionally, Huang et al. [60] isolated two polysaccharide–peptide fractions from *P. citrinopileatus* that reduced ALT, AST, and intracellular triglyceride levels. In hepatocyte models of HepG2 cells, cell viability was highly maintained when treated with these fractions, indicating mitigation of liver damage [60]. Additionally, Huang et al. found that the polysaccharide–peptide fractions increased until 66.35% of the superoxide dismutase (SOD) activity, showing the capacity to protect hepatocytes against oxidative stress. In both cases, polysaccharides interact with receptors to activate Nrf2 and, as a consequence, SOD increased, and M1 macrophages were modulated to M2; therefore, polysaccharides and peptides can act synergistically to attenuate hepatic injury [60,83].

The potential applications of *Pleurotus* spp. proteins, protein hydrolysates, and peptides in human health have been investigated mostly through in silico approaches [46,84] and in vitro systems [17,27,44,46,51]. In vivo models of these complexes have been carried out, but they remain insufficiently explored, representing a significant research gap [27,61]. Therefore, further research is needed to determine if these promising biological activities can be reproduced under physiological conditions and to establish their applicability to human health. In particular, understanding the behavior of these proteins and peptides during gastrointestinal digestion is essential, as enzymatic hydrolysis and peptide stability can modify their bioaccessibility, bioavailability, and biological activity.

### 5.6. Antimicrobial Activity of Bioactive Proteins, Hydrolysates and Peptides from Pleurotus spp.

Bioactive peptides from the *Pleurotus* genus have also attracted considerable attention due to their antimicrobial and antibiofilm properties [11,35]. The mechanism of action of antimicrobial peptides involves multiple cellular targets, which reduce microbial resistance across a broad spectrum of microorganisms. This is particularly relevant for multidrug-resistant pathogens [18,59]. Antimicrobial peptides are short peptides (2 to 20 amino acids) with hydrophobic amino acids such as Pro, Val, Trp, and Phe, which facilitate their interaction with microbial membranes and disrupt the cell membrane, leading to loss of integrity and cell death [18,85]. Furthermore, these peptides interfere with biofilm formation, which is a complex process involving initial attachment, maturation, and persistence of microbial communities [18]. Peptides derived from *Pleurotus* spp. disrupt quorum sensing signaling routes in Gram-positive and Gram-negative bacteria by interacting with autoinducer molecules. Consequently, microbial communication is inhibited, biofilm formation is limited, and survival of persistent cells is reduced [18,46].

Gangwar et al. [18] demonstrated that proteins obtained from *Pleurotus* spp. exhibited inhibitory activity against a broad spectrum of dental caries pathogens, even if the microorganisms were resistant to antibiotics, such as *Staphylococcus aureus* (methicillin-resistant strains, MRSA) and *Candida albicans* resistant to nystatin. According to the authors, 5.0 μg of *P. ostreatus* protein was enough to inactivate *C. albicans,* while nystatin, a standard antifungal, showed no detectable activity against this fungus. They argued that the proteins of *Pleurotus* spp. rely on non-ergosterol-binding mechanisms; ergosterol is the common target of antifungals. Ventura-García et al. [46] isolated in silico peptides from protein hydrolysates and, by molecular docking, predicted that TSMQSSL, TSQESY, QEGQKL, PGSHPV and GRNSL peptides could have anti-quorum sensing activity against some Gram-positive bacteria. However, these activities have not been experimentally proven against live bacteria. In addition, Lu et al. [86] found three protein compounds from *P. ostreatus* that showed antifungal activity against *C. albicans* with a MIC value of 500 μg/mL, the same as the standard antifungal Fluconazole.

### 5.7. Applications of Bioactive Proteins, Hydrolysates and Peptides from Pleurotus spp. in the Food Industry

In the food industry, proteins and peptides derived from *Pleurotus* spp. have shown interesting physicochemical properties that can influence processing performance, texture, shelf life, product stability, and flavor [11,39]. Rheological properties play a crucial role in food processing and in maintaining texture stability, commonly reflected in apparent viscosity, which reflects a solution’s flow and deformation characteristics [39]. According to Shi et al. [39], protein solutions derived from *Pleurotus* spp. normally present non-Newtonian behavior, where viscosity decreases with increasing shear rate, reducing resistance to flow [43]. This behavior is associated with the progressive disruption and protein orientation in the direction of flow when shear is applied [39]. These authors found that increasing *P. eryngii* protein concentration in a solution enhances viscosity due to increased shear stress and an expanded hysteresis loop, driven by the stabilization of hydrophobic interactions and disulfide bonds, which promote protein aggregation and gel formation [39]. In another context, Fan et al. [33] found that when they increased protein concentration from 5 to 20 mg/mL in a Pickering emulsion, the volume-weighted mean diameter of droplets (d_4,3_) decreased from 90 µm to 20 µm and emulsions with oil-volume fractions of 0.1 to 0.5 remained stable after 4 days of storage at room temperature. This rheological behavior is linked to emulsification, moisture preservation, and maintaining food texture stability, affecting food sensory and technological characteristics [14,39,87].

Extraction and processing conditions can influence rheological characteristics. González et al. [11] found that as pH increased, *P. ostreatus* protein solubility also increased because proteins gain a net negative charge, enhancing electrostatic repulsion and helping protein dispersion in aqueous systems. Electrostatic repulsion could be measured by zeta potential, where high absolute values (close to ±30 mV) indicate adequate dispersibility, while lower values suggest aggregation or flocculation of proteins [1,33]. In contrast, Shi et al. [39] pointed out that at low pH values (near the isoelectric point), electrostatic repulsion decreases, and disulfide bond formation increases, causing protein aggregation with a more ordered and stable structure, and thereby enhancing gel strength and structural integrity. At neutral pH, higher sulfhydryl content can lead to more flexible and less crosslinked protein conformations, resulting in weaker complex formation and lower viscosity [30,39,88]. Fan et al. [33] reported that protein from *P. tuoliensis* increased its solubility from 0 to over 70%, as pH increased from 4 to 9. Similarly, Wu et al. [30] observed that the solubility of 50–100 kDa protein from *P. eryngii* was improved from 15 g/100 g to 70 g/100 g when pH was adjusted from 4 to 11. In addition, these authors found that the emulsifying activity and stability of that protein increased from 20 g/mL to over 60 g/mL and from 12 min to more than 50 min, respectively, when pH in solutions was increased from 4 to 10.

In contrast, viscosity decreases with increasing temperature due to enhanced molecular mobility, which facilitates chain movement and reduces intermolecular resistance [39]. Enzymatic hydrolysis modifies protein structure from *Pleurotus* spp., since particle size is reduced and the secondary and tertiary conformations are transformed into more flexible arrangements, enhancing diffusion at oil–water or air–water interfaces, and improving functional properties such as emulsification and stability [43]. For example, Khapudang et al. [1] reported that the zeta potential of *P. ostreatus* increased its negativity from −5.95 mV to −19.8 mV when the extraction method was shifted from alkali isoelectric precipitation to enzyme-assisted extraction, and particle size decreased from 621.8 nm to 288.5 nm, indicating enhanced stability.

Some authors have also investigated *Pleurotus* spp. protein–polysaccharide interactions [12]. These interactions involve electrostatic forces, hydrogen bonds and hydrophobic interactions that can improve the structural and functional properties of food systems [12]. When both protein and polysaccharide have similar charges, electrostatic repulsion may induce phase separation, promoting gelation (also depending on pH as mentioned before) [12,89]. Additionally, these interactions induce protein conformational changes, such as variations in α-helix and β-turn content that can confer softness and flexibility to the system, and β-sheet content that can stabilize and strengthen gels [12,90,91]. These changes improve with rising temperature [12,90,91]. Liu et al. [12] found an increase in gelatinization temperature, storage modulus, loss modulus, complex modulus, and viscosity, and a decrease in gelation time when *P. ostreatus* protein was added to 1% carrageenan gels.

Additionally, specific proteins from *Pleurotus*, such as hydrophobins, exhibit remarkable interfacial activity due to their amphiphilic nature and can self-assemble at hydrophobic–hydrophilic interfaces, forming stable films, stabilizing foams and emulsions, and modifying surface wettability [37]. Kulkarni et al. [37] isolated a hydrophobin from *P. ostreatus* of 10–17 kDa that reduced the contact angle of water by 14.4% due to its interactions with the Teflon surface, increasing the hydrophilicity of systems.

#### 5.7.1. Umami Effect of Peptides from *Pleurotus* spp.

In the food flavor context, umami is recognized as the fifth basic taste and plays a fundamental role in palatability. This taste is present in protein-rich foods, such as mushrooms from the genus *Pleurotus*, contributing to their characteristic flavor profile, and is attributed to free amino acids, nucleotides, organic acids, and, above all, peptides [48,52,59]. These peptides produce their sensory effects through interactions with specific umami taste receptors, such as the heterodimer T1R1/T1R3, and the metabotropic glutamate receptors mGluR1 and mGluR4 [48,52]. The T1R1/T1R3 receptor complex is the principal mediator of umami perception, where T1R1 is involved in taste identification, while T1R3 provides a binding domain with high affinity for peptides [48]. Molecular interactions between peptides and these receptors are produced by hydrogen bonding and hydrophobic interactions, enabling stable ligand–receptor complexes [48,52].

The umami potential of peptides is strongly influenced by their molecular weight, amino acid composition, and structural conformation [48]. Low-molecular-weight peptides, particularly those with two or three amino acids, exhibit stronger umami intensity, although longer peptides can also contribute depending on their conformation and sequence [48]. On the other hand, glutamic acid (Glu) and aspartic acid (Asp) are key umami-contributing amino acids, acting as active sites inside peptide sequences regardless of their position [48,92,93]. Additionally, amino acid residues such as asparagine (Asn) and glutamine (Gln) could enhance this effect [48,92,93]. Wang et al. [48] identified four umami peptides from *P. citrinopileatus* (LLVTVEDELRV, LDDWR, ELFEPT, and ENLLEK) whose umami properties were confirmed through sensory evaluation and electronic tongue analysis. Molecular docking also demonstrated the binding affinity of these four umami peptides to the T1R1/T1R3 complex through hydrogen bonds. Wang et al. [52] identified three umami-enhancing peptides (DRAFR, LDFR, and VLRDF) from *P.* that enhance the flavor of a monosodium glutamate solution. Furthermore, molecular docking showed that the identified umami peptides possessed interaction sites at Asp218, Asp108, Cys50, Gly49, Arg151, Arg357, and Leu308, which could relate to umami receptors.

Beyond umami, certain peptides derived from *Pleurotus* spp. also contribute to kokumi taste, a flavor-enhancing sensation associated with mouthfulness and continuity [58,93]. This effect is mediated by the calcium-sensing receptor (CaSR) located in the taste buds of the tongue, which can be activated by specific peptides such as γ-glutamyl peptides. These compounds do not have a distinct taste themselves but enhance existing flavors, including umami and saltiness, making them valuable for reducing sodium content in food formulations [58,93]. For instance, Xia et al. [58] generated *P. geesteranus* protein hydrolysates modified with γ-glutamyl transpeptidase from *Bacillus amyloliquefaciens* to produce γ-glutamyl peptides that enhanced umami and saltiness intensity of chicken soup, measured by an electronic tongue.

#### 5.7.2. *Pleurotus* spp. and Pleurotus Proteins as Components of Meat Analogs

In recent years, concerns regarding meat intake have increased due to its effects on human health and the sustainability issues associated with its production [94]. *Pleurotus* mushrooms contain approximately 19–35% dw of protein and normally provide all essential amino acids, as well as vitamin C and some B vitamins such as thiamine, niacin and riboflavin, in addition to their desirable physicochemical and sensory characteristics mentioned before [94]. In this sense, *Pleurotus* spp. and *Pleurotus* proteins have attracted attention as ingredients for meat products and meat analogs to improve nutritional quality, increase dietary fiber content, enhance organoleptic properties, and reduce caloric, purine, and sodium contents [26,94]. Zhao et al. [26] incorporated 3% *P. eryngii* protein into chicken mince products, reporting an increase in the β-turn and β-sheet structures within the protein gel matrix, improving gel stabilization, texture, and palatability. Furthermore, the incorporation of *P eryngii* proteins improved sensory quality and reduced lipid oxidation.

Most of the remaining evidence of *Pleurotus* spp. meat analogs involves the use of fresh or dried whole mushroom, not isolated protein or peptides, reflecting the combined contribution of all mushroom components. Partial replacement of beef, pork, chicken, and fish with *Pleurotus* spp. has proven to be a viable strategy for developing meat products with high fiber and moisture contents, lower fat and caloric values, as well as lower production costs [95,96,97,98,99,100]. Moreover, the incorporation of these mushrooms has been associated with reduced pH, lightness (L*), and shear force values [95,96,97,98,99,100]. For instance, Bermudez et al. [97] replaced 5% of beef in patties with *P. djamor,* observing a decrease in the cooking loss from approximately 22% to 10%, an increase in dietary fiber content from 0% to 1.2%, and a reduction in fat content from 3.6% to 3.0% without affecting protein levels. If’all et al. [98] substituted 33% of beef with fresh *P. ostreatus* in meatballs, reducing fat content from 15.8 to 7.2% while increasing crude fiber from 0.80 to 3.10%. Likewise, Yahya and Ting [99] replaced 60% of chicken meat with *Pleurotus sajor-caju* in sausages, increasing fiber content from 0.6% to 1.5% and decreasing crude fat from 4.43% to 3.46%. Pachekrepapol et al. [101] supplemented salmon and striped catfish burgers with 15% (w/w) of *P. ostreatus* and found that the mushroom addition reduced thiobarbituric acid reactive substances values, improved thawing loss, and increased cooking yield during storage at −18 °C.

Maseko et al. [102] experimented with a distinct line using mushroom mycelia, and they reported that it could contain up to 85% dw of protein and a fibrous structure; hence, *Pleurotus* mycelium is a promising ingredient for meat products or meat analog formulations [103]. Stephan et al. [104] developed vegan boiled sausages supplemented with *Pleurotus sapidus* mycelia and observed improvements in texture (strength and hardness), flavor scores from 3.7 to 6.1 on a 9-point hedonic scale, and texture scores from 6.5 to 7.2, while maintaining these characteristics during four weeks of storage. Similarly, Chawasirikhunthol et al. [105] formulated pork patty substitutes with 34.4% dried *P. pulmonarius* mycelium, increasing protein content from 0.049 to 0.084% (w/w) without significant differences in taste, texture, color, or odor.

## 6. Conclusions

Proteins, protein hydrolysates, and peptides derived from the *Pleurotus* genus, particularly low-molecular-weight fractions, show many bioactivities that can be altered by processing conditions (temperature, pH, and extraction method). The greatest bioactivities of these compounds are their potential ability to mitigate and prevent oxidative stress due to their capability to modulate key metabolic pathways, to scavenge free radicals, and to protect biological systems. These activities could be relevant to processes associated with systemic inflammation, arterial hypertension and cardiovascular problems, immune suppression, and some types of cancer through autophagy, apoptosis, and cell-cycle arrest. However, further in vivo and human studies are needed to understand the mechanisms of action and toxicity. Moreover, these *Pleurotus* spp. derivatives present hepatoprotective and lipid-regulating activities. In food systems, the antioxidant properties are particularly valuable, as they can inhibit lipid peroxidation and improve product stability and shelf life. In addition, these compounds have antimicrobial effects that target resistant microorganisms and disrupt biofilm formation. Furthermore, *Pleurotus*-derived proteins could provide a versatile option for designing food systems with desired rheological and structural properties such as stability, viscosity, and solubility. Bioactive peptides and free amino acids from these mushrooms can interact with taste receptors and enhance flavor perception, being an alternative to artificial flavors. Therefore, *Pleurotus*-derived proteins, protein hydrolysates, and peptides could represent promising compounds with potential applications in human health and in food formulation and preservation, as natural alternatives to conventional preservatives and synthetic food antioxidants, as well as stabilizers and flavorings. Furthermore, due to their high protein and fiber content, *Pleurotus* mushrooms and derived proteins have great potential for application in meat products and meat analogs.

## Data Availability

No new data were created or analyzed in this study. Data sharing is not applicable to this article.
